# Genome-wide analysis of OSCA gene family members in *Vigna radiata* and their involvement in the osmotic response

**DOI:** 10.1186/s12870-021-03184-2

**Published:** 2021-09-07

**Authors:** Lili Yin, Meiling Zhang, Ruigang Wu, Xiaoliang Chen, Fei Liu, Baolong Xing

**Affiliations:** 1 College of Life Science, Shanxi Datong University, Datong, 037009 People’s Republic of China; 2 Beijing Academy of Forestry and Pomology Sciences, Beijing, 100093 People’s Republic of China; 3 School of Landscape and Ecological Engineering, Hebei University of Engineering, Handan, 056038 People’s Republic of China; 4 School of Medicine, Shanxi Datong University, Datong, 037009 People’s Republic of China; 5 High Latitude Crops Institute, Shanxi Agricultural University, Datong, 037008 People’s Republic of China

**Keywords:** Mung bean (*Vigna radiata*), OSCA gene family, Evolutionary analysis, Expression patterns, Abiotic stress

## Abstract

**Background:**

Mung bean (*Vigna radiata*) is a warm-season legume crop and belongs to the papilionoid subfamily of the Fabaceae family. China is the leading producer of mung bean in the world. Mung bean has significant economic and health benefits and is a promising species with broad adaptation ability and high tolerance to environmental stresses. OSCA (hyperosmolality-gated calcium-permeable channel) gene family members play an important role in the modulation of hypertonic stress, such as drought and salinity. However, genome-wide analysis of the OSCA gene family has not been conducted in mung bean.

**Results:**

We identified a total of 13 *OSCA* genes in the mung bean genome and named them according to their homology with *AtOSCAs*. All the *OSCAs* were phylogenetically split into four clades. Phylogenetic relationship and synteny analyses showed that the *VrOSCAs* in mung bean and soybean shared a relatively conserved evolutionary history. In addition, three duplicated *VrOSCA* gene pairs were identified, and the duplicated *VrOSCAs* gene pairs mainly underwent purifying selection pressure during evolution. Protein domain, motif and transmembrane analyses indicated that most of the VrOSCAs shared similar structures with their homologs. The expression pattern showed that except for *VrOSCA2.1*, the other 12 *VrOSCA*s were upregulated under treatment with ABA, PEG and NaCl, among which *VrOSCA1.4* showed the largest increased expression levels. The duplicated genes *VrOSCA2.1*/*VrOSCA2.2* showed divergent expression, which might have resulted in functionalization during subsequent evolution. The expression profiles under ABA, PEG and NaCl stress revealed a functional divergence of *VrOSCA* genes, which agreed with the analysis of cis-acting regulatory elements in the promoter regions of *VrOSCA* genes.

**Conclusions:**

Collectively, the study provided a systematic analysis of the VrOSCA gene family in mung bean. Our results establish an important foundation for functional and evolutionary analysis of *VrOSCAs* and identify genes for further investigation of their ability to confer abiotic stress tolerance in mung bean.

**Supplementary Information:**

The online version contains supplementary material available at 10.1186/s12870-021-03184-2.

## Background

Under natural environmental conditions, plants are subjected to many types of stress. Osmotic stress caused by drought and salinity is one of the key stress factors affecting plant growth and yield [1]. Osmotic stress usually disrupts the plant osmotic balance and finally causes damage to the cell membrane system [2]. In many agricultural ecosystems, water scarcity and drought could induce phosphorus deficiency, which limit crop yield significantly [3]. It has been reported that salinity stress impairs normal metabolic pathways, such as photosynthesis, respiration, mineral assimilation and biomass accumulation, thereby contributing considerably to reduced crop production [1, 4, 5]. Previous studies have found that plant responses to stress mainly include the perception and transmission of signals through various pathways and the regulation of stress-responsive gene expression, resulting in physiological and morphological modifications to resist stress [6–8]. These changes are mainly manifested in the enhancement of proline, betaine and sugar synthesis, which helps to the maintenance of tissue water content, and the up-regulation of key antioxidant enzymes activity to reduce the oxidation of proteins and lipids by reactive oxygen species [9–11]. During signal perception, calcium is an important second messenger in the signal transduction pathway when plants respond to stress [12, 13]. Under osmotic stress, plants induce a rapid intracellular increased concentration of free calcium ions, thereby inducing the expression of many stress-related genes to regulate plant tolerance to osmotic stress [7, 14, 15]. The increased intracellular concentration of calcium ions is mainly regulated by calcium transport systems such as calcium channels and calcium pumps [16]. Previous studies showed that stimuli-gated Ca^2+^ permeable channels served as osmosensors in bacteria and animals [17, 18], which indicated that there might be specific calcium permeable channels that function as osmosensors in plants.

In plants, OSCA is calcium nonselective cation channel protein and receptor protein for hypertonic stress [19–21]. Studies of the functional domain show that the OSCA gene family contains a calcium-dependent channel domain (DUF221) that may participate in osmotic adjustment [22, 23]. In rice, the entire OSCA gene family is characterized by the presence of a conserved DUF221 domain, which functions as an osmotic-sensing calcium channel [24]. In *Arabidopsis*, OSCA1, a hyperosmolality-gated calcium-permeable channel, was characterized as an osmosensor and mediated osmotic-stress-evoked Ca^2+^ concentration increases [20]. Studies have shown that the maize gene *ZmOSCA2.4* could enhance drought tolerance in transgenic *Arabidopsis* [25]. OSCA family members play a crucial role in plant resistance to osmotic stress. Therefore, it is important to identify and study potential genes for breeding osmotic stress-resistant varieties. Predecessors have systematically identified and analyzed the OSCA gene family in dicotyledons, including *A. thaliana* and soybean, and monocotyledon rice [20, 24, 26]. However, genome-wide analysis of the OSCA gene family has not been conducted in mung bean.

Mung bean (*Vigna radiata* (L.) R. Wilczek, 2n = 2 × =22) belongs to the papilionoid subfamily of Fabaceae and is always grown in poor-soil regions because of broad adaptation ability and high tolerance to stress. Mung bean seeds are rich in protein and contain higher levels of folic acid and iron than most other legumes [27]. Completion of the mung bean genome sequence has allowed an opportunity to systematically research the OSCA gene family in mung bean [28]. In the present study, we identified putative OSCA gene family members in mung bean and analyzed their phylogeny, syntenic relationships, conserved motifs, transmembrane regions (TMs) and promoter regions containing cis-regulatory elements responsive to abiotic stress. In addition, we studied the expression profiles of *OSCAs* following treatment with PEG, NaCl and ABA. These findings will facilitate further research on the biological function of this gene family and provide putative gene targets for the cultivation of genetically modified osmotic stress-resistant plants.

## Results

### Genome-wide identification of *OSCA* gene family members in mung bean

The hidden Markov model (HMM) of the DUF221 domain (Pfam accession number: 02714) was used to search against the mung bean genome. Ultimately, a total of 13 *VrOSCA* genes were identified in mung bean and named according to the *Arabidopsis* orthologues (Table 1). Among the 13 genes, 12 *VrOSCA* genes were distributed randomly on all 11 chromosomes except chromosomes 2, 8 and 10, while *VrOACA2.5* was located on scaffold_100. The number of amino acids of the identified VrOACAs varied from 592 (VrOACA2.2) to 880 (VrOACA4.1). The molecular weight (MW) of the VrOSCA proteins varied from 67.16 (VrOACA2.2) to 99.16 kDa (VrOACA4.1) and the isoelectric points (pI) ranged from 6.28 (VrOACA4.1) to 9.44 (VrOACA2.5).

**Table 1:** Detailed information for 13 *VrOSCA* genes in the *V. radiata* genome

Gene Name	Gene Identifier	Chromosome	Gene length (bp)	Protein length (aa)	ORF (bp)	Isoelectric Point	Molecular Weight (KDa)	Clade
*VrOACA1.1*	Vradi07g26860	7	6435	775	2328	8.91	88.17	1
*VrOACA1.2*	Vradi10g01410	10	7609	773	2322	9.22	88.68	1
*VrOACA1.3*	Vradi06g03460	6	5939	760	2283	9.08	87.48	1
*VrOACA1.4*	Vradi03g00620	3	7957	640	1923	6.68	72.55	1
*VrOACA1.5*	Vradi11g08350	11	6738	863	2592	9.06	98.71	1
*VrOACA2.1*	Vradi06g14350	6	8460	721	2166	8.53	81.83	2
*VrOACA2.2*	Vradi05g11970	5	16,125	592	1779	8.37	67.16	2
*VrOACA2.3*	Vradi01g07680	1	11,006	709	2130	8.52	80.50	2
*VrOACA2.4*	Vradi04g08970	4	6275	637	1914	6.71	72.90	2
*VrOACA2.5*	Vradi0100s00520	Scaffold_100	5251	671	2016	9.44	76.95	2
*VrOACA2.6*	Vradi05g17480	5	10,518	600	1803	9.03	67.43	2
*VrOACA3.1*	Vradi10g09440	10	4444	728	2187	9.36	82.44	3
*VrOACA4.1*	Vradi07g01560	7	7051	880	2643	6.28	99.16	4

### Phylogenetic analysis of the *OSCA* gene family in mung bean

**Fig. 1:**
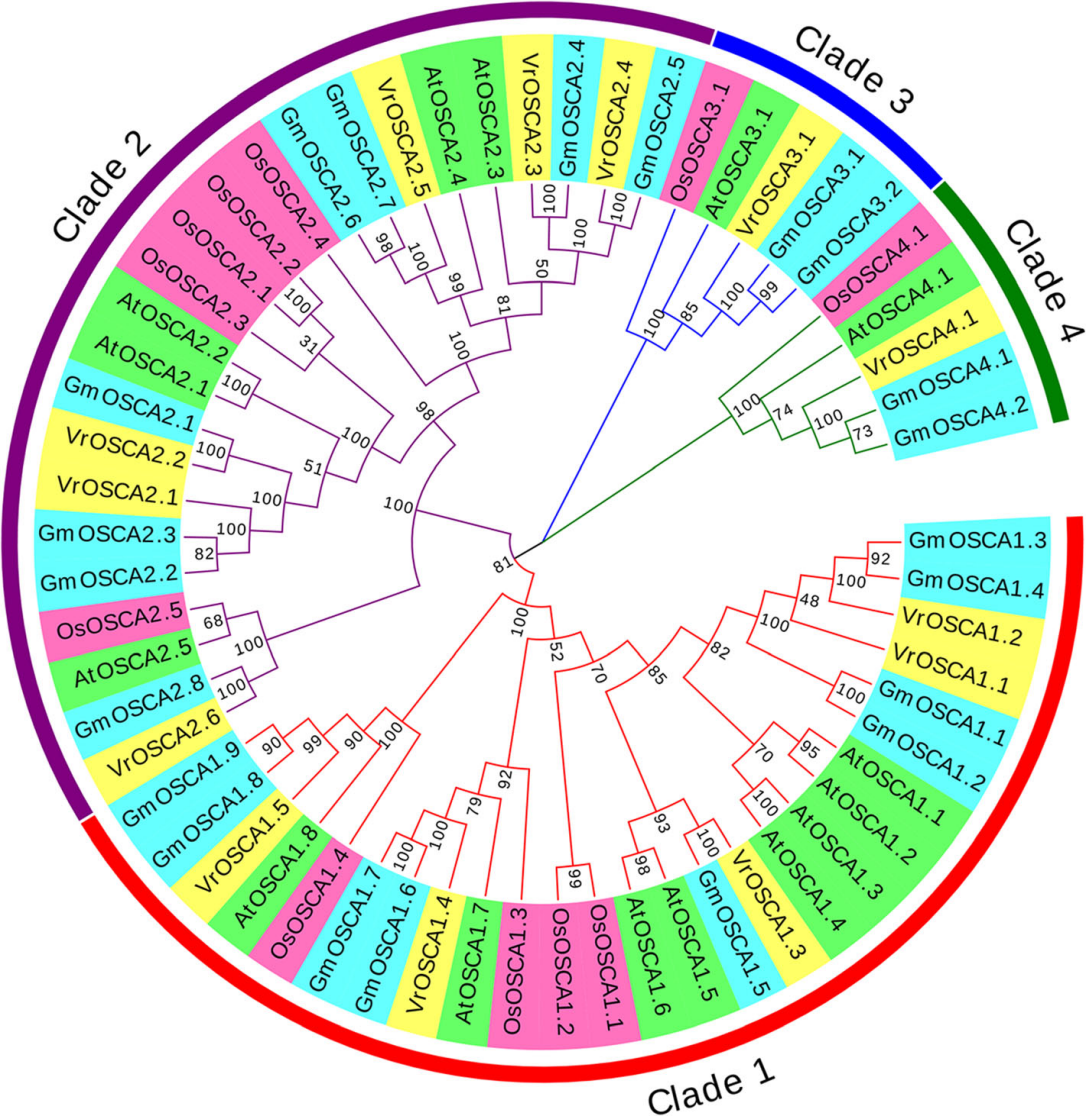
Phylogenetic tree of the *OSCA* gene family in mung bean, soyean, *Arabidopsis* and rice. The neighbor-joining tree was generated through the MEGA7 program using the amino acid sequences of the OSCA proteins by the neighbor-joining (NJ) method, with 1000 bootstrap replicates. The four major phylogenetic clades (1 to 4) are labelled and the OSCAs from different species are denoted by different colored backgrounds

To elucidate the phylogenetic relationships of OSCA proteins in mung bean, *Arabidopsis*, soybean and rice, a phylogenetic tree based on the alignment of 60 full-length OSCA protein sequences was built (Additional file 1). The 60 OSCA proteins were classified into four major groups, clades 1, 2, 3, and 4. Clades 1 and 2 contained more members than clades 3 and 4 (Fig. 1). Phylogenetic analysis results showed that the OSCA gene family underwent a similar evolutionary history when comparing between the mung bean, *Arabidopsis*, soybean and rice genomes. Moreover, OSCA proteins derived from mung bean had a higher similarity to those from soybean (Fig. 1), demonstrating a closer phylogenetic relationship between mung bean and soybean since both belong to the Fabaceae family.

### Collinearity analysis of *OSCA* genes in mung bean, *Arabidopsis*, soybean and rice

**Fig. 2:**
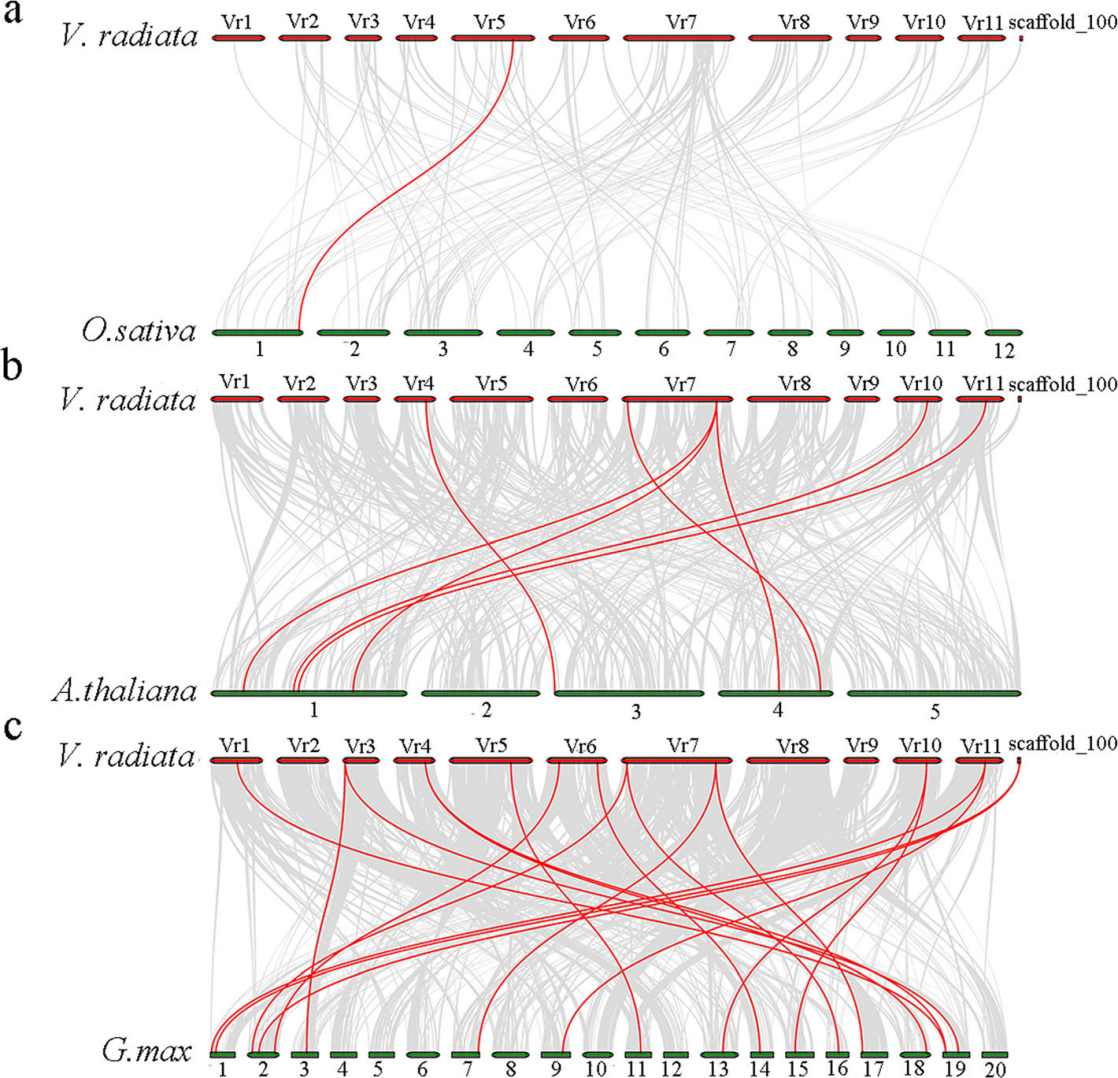
Schematic diagram of synteny analysis of *OSCA* genes between mung bean and other plant species. The species names with the prefixes *‘V. radiata’, ‘O. sativa’, ‘A. thaliana’* and *‘G. max’* indicate *Vigna radiata*, *O. sativa, A. thaliana* and *G. max,* respectively. Grey lines in the background are the duplication events between the mung bean and other plant genomes, while the red lines indicate the syntenic *OSCA* gene pairs. The chromosome number is labelled at the top or bottom of each chromosome. Red and green bars represent the chromosomes

Comparative genomics analyses of gene collinearity reveal homologous gene functions and phylogenetic relationships between species. Thus, we analyzed the collinearity relationship of *VrOSCA* genes with three representative species, including one monocot (rice) and two dicots (*Arabidopsis* and soybean). We found that the *OSCA* genes of mung bean had the most homologous gene pairs with the *OSCA* genes of *Glycine ma* (17), followed by *Arabidopsis* (7) and *O. sativa* (1) (Fig. 2, Additional file 2), indicating that in comparison with *Arabidopsis* and rice, mung bean *OSCA* genes show a closer phylogenetic relationship with soybean *OSCA* genes. This result was consistent with the phylogenetic analysis (Fig. 1), affirming the accuracy of our analysis. Some *VrOSCAs* (*VrOSCA1.1, − 1.4, − 1.5, − 2.4, − 2.5, − 3.1* and *− 4.1*) were found to be associated with two syntenic gene pairs in mung bean and soybean (Additional file 2). These genes may play a crucial role during evolution. Meanwhile, no collinear segments of *VrOSCA1.2* and *VrOSCA2.2* were found in the genomes of mung bean and soybean (Additional file 2). The results indicate that large-scale expansion of *OSCAs* probably occur before the mung bean-soybean division, and certain *VrOSCAs* might have originated from duplication of the mung bean genome after the phylogenetic divergence of mung bean.

### Gene duplication of *VrOSCAs* in mung bean

**Fig. 3:**
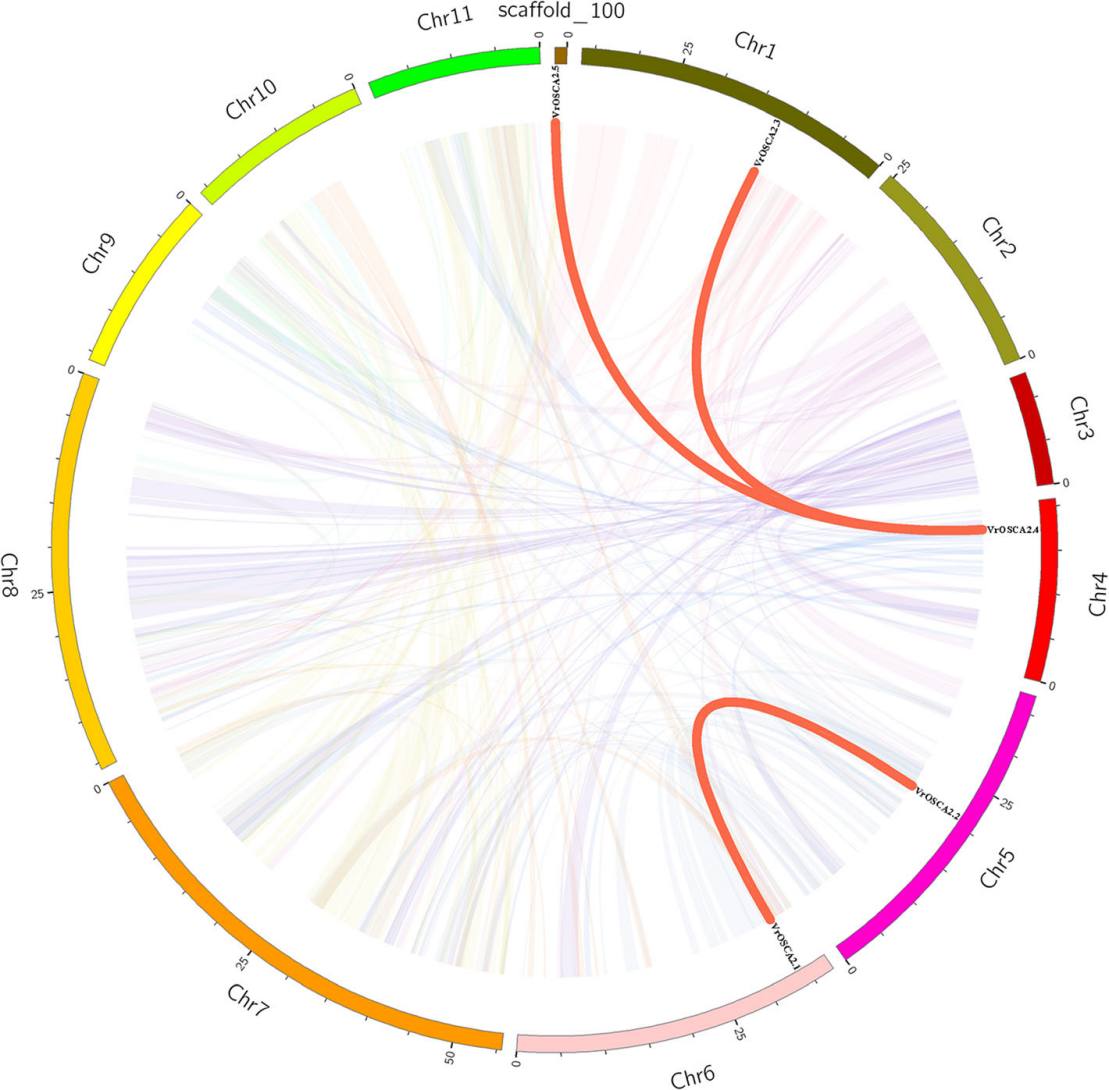
Schematic representations of segmental duplications of *OSCA* genes. Colorized lines indicate all synteny blocks between each chromosome and the thick red lines indicate duplicated OSCA gene pairs. The chromosome number is shown at the bottom of each chromosome. The scale bar marked on the chromosome is the length of the chromosome (Mb).

To better understand the evolutionary relationship, gene duplication events were analyzed to elucidate the expansion patterns of the *OSCA* genes in mung bean. Three segmental duplication events with five *OSCAs* were identified, which were located on duplicated segments on chromosomes 1, 4, 5, 6 and scaffold_100 (Fig. 3). Moreover, the Ka/Ks ratio of the duplicated *VrOSCA* gene pairs was calculated to evaluate the molecular evolution. All of the Ka/Ks ratios were less than 1 (Table 2).

**Table 2:** Ka/Ks analysis for duplicated gene pairs of *OSCAs* in mung bean

Duplicated Gene 1	Duplicated Gene 2	Type of duplication	Ka	Ks	Ka/Ks	Purifying Selection
*VrOACA2.1*	*VrOACA2.2*	segmental	0.1478	0.7539	0.1960	Yes
*VrOACA2.3*	*VrOACA2.4*	segmental	0.1397	0.6934	0.2015	Yes
*VrOACA2.4*	*VrOACA2.5*	segmental	0.3050	2.2950	0.1329	Yes

### Conserved domain, motif and TM analyses of VrOSCA proteins

**Fig. 4:**
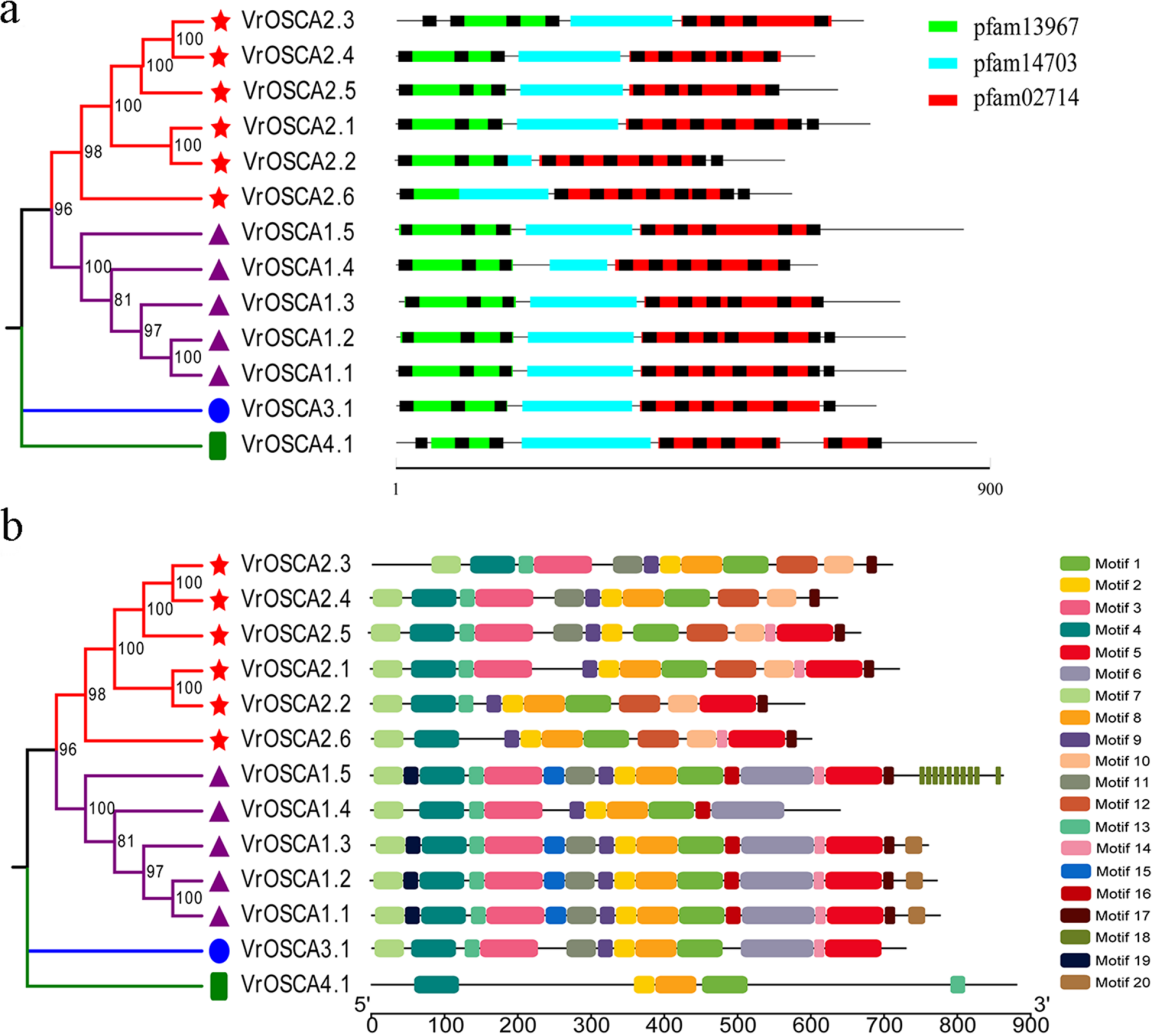
Distribution of protein domains, TMs and conserved motifs in VrOSCA proteins. Grey lines represent amino acid sequences. **a** Schematic diagram of functional domains and TMs. The black rectangles indicate the TMs and the colored rectangles represent the protein domains. **b** Schematic diagrams of all motifs in VrOSCA proteins. Different motifs are annotated by boxes of different colors and numbered 1–20. The regular expression sequence of each motif is listed in Additional file 5

Analysis of the protein conserved domains of VrOSCAs revealed that most VrOSCAs contained three domains: late exocytosis domain (pfam13967), cytosolic domain of 10 TM putative phosphate transporter (pfam14703, DUF4463) and calcium-dependent channel domain (pfam02714, DUF221), while VrOSCA4.1 contained four domains, including two DUF221 protein domains, as shown in Fig. 4a and Additional file 3. Notably, the pfam13967 and pfam02714 protein domain are located at the N-terminus and C-terminus of all VrOSCAs, respectively, and the pfam14703 protein domain is located in the middle of pfam13967 and pfam02714 domains (Fig. 4a). These results indicate that the three domains are relatively conserved in the VrOSCA family. Meanwhile, it was discovered that the pfam13967 and pfam02714 protein domains contained a different number of TMs, while no TMs were detected in the pfam14703 protein domain in any of the VrOSCAs (Fig. 4a, Additional file 4). All the VrOSCAs contained at least eight TMs (Fig. 4a, Additional file 4).

To further explore the potential function of VrOSCAs, we detected additional conserved motifs using the Multiple EM for Motif Elicitation (MEME) tool, and a total of 20 conserved motifs were detected (Fig. 4b, Additional file 5). Notably, all clades contained motifs 1, 2 and 4 (Fig. 4b), indicating that all genes perform the three functions. Among them, motif 1 and motif 2 were located in the calcium-dependent channel domain, and motif 4 was located in the late exocytosis domain (Fig. 4). Some conserved domains were restricted to specific clades. For example, motif 16 and motif 12 were only detected in clade 1 and clade 2, respectively (Fig. 4b), which indicated functional differences between clade 1 and clade 2. We also observed different motifs within the same clade (Fig. 4b), suggesting that there were different mechanisms of action within each clade. For example, VrOSCA1.4 in clade 1 lacked motifs 5, 14, 15, 17 and 19, whereas the other four VrOSCAs (VrOSCA1.1, − 1.2, − 1.3 and − 1.5) in clade 1 contained these motifs (Fig. 4b). This phenomenon was also observed in other clades. VrOSCA4.1 in clade 4 contained the fewest motifs (Fig. 4b). The results of the conserved motif analysis were generally consistent with the phylogenetic analysis.

### Expression of *VrOSCA*s under ABA and abiotic stress

**Fig. 5:**
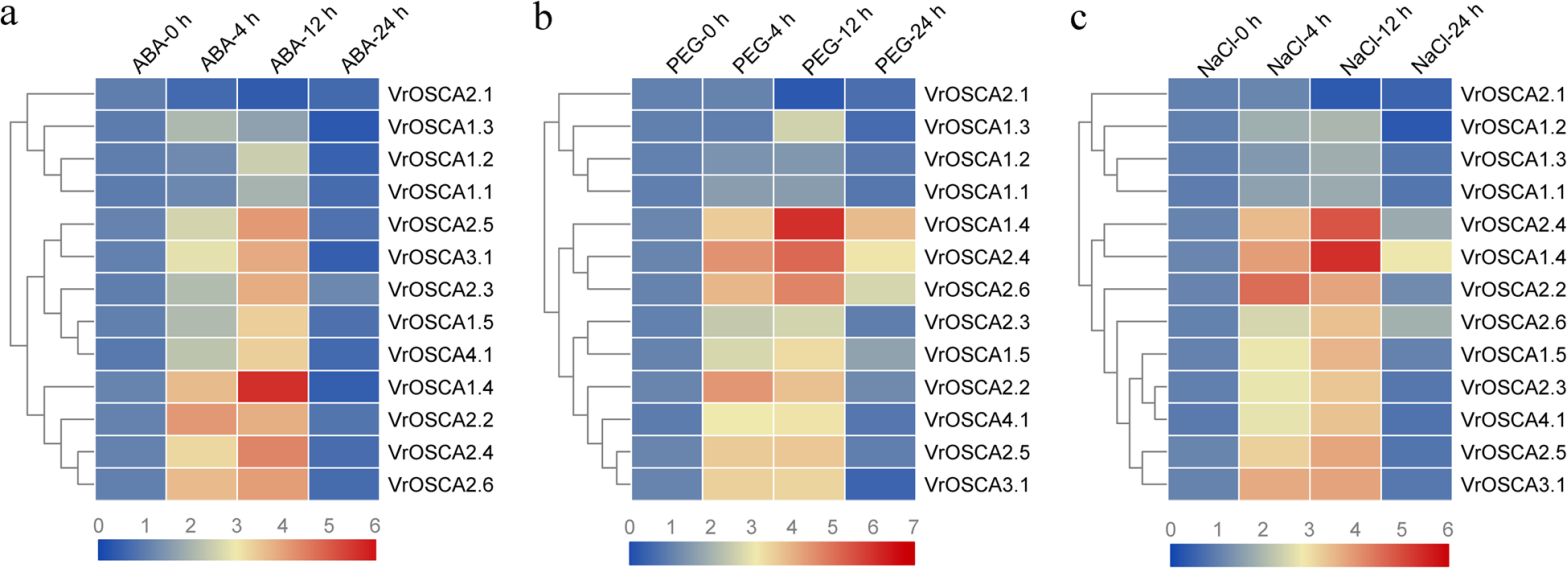
Heat map of the expression of 13 *VrOSCA* genes under ABA (**a**), PEG (**b**) and NaCl (**c**) treatment. The color scale at the bottom of the picture represents log2 expression values, where blue indicates a low level and red indicates a high level of transcript abundance. Gene names are listed on the right. The expression-based hierarchical clustering of genes is shown

PEG and NaCl stress may cause similar cellular damage and lead to osmotic stress [29]. Plants adapt and respond to drought and salt stress by inducing the expression of a range of genes. ABA is an important plant hormone that regulates the expression of stress-responsive genes in plants [30]. We studied the expression profiles of the 13 *VrOSCA* genes following treatment of mung bean with ABA, PEG and NaCl for 4 h, 12 h and 24 h. Analysis of the expression profiles showed that except for *VrOSCA2.1*, the other 12 *VrOSCA* genes were upregulated by ABA, PEG and NaCl treatment. *VrOSCA2.1* was significantly downregulated by ABA, PEG and NaCl treatment (Fig. 5, Additional file 6). The expression patterns of all the upregulated genes increased at 4 h or 12 h and then decreased at 24 h of stress. The relative expression values of *VrOSCA1.4*, *− 2.2*, *− 2.3*, *− 2.4*, *− 2.5*, *− 2.6*, *− 3.1* and *− 4.1* genes were relatively higher than those of *VrOSCA1.1*, *− 1.2* and *− 1.3* genes under the three types of osmotic stress (Fig. 5, Additional file 6). Additionally, following ABA treatment, genes expression increased by a factor of more than 10-fold in *VrOSCA1.4, − 2.2*, *− 2.3*, *− 2.4*, *− 2.5*, *− 2.6* and *− 3.1* genes when compared to expression at 0 h of treatment. Following PEG treatment, the expression of *VrOSCA1.4, − 2.2*, *− 2.4*, *− 2.5*, *− 2.6* and *− 3.1* genes increased by a factor of more than 10-fold when compared to expression at 0 h of treatment. Following NaCl treatment, the expression of *VrOSCA1.4*, *− 1.5*, *− 2.2*, *− 2.4*, *− 2.5* and *− 3.1* genes increased by a factor of more than 10-fold when compared to expression at 0 h of treatment (Additional file 6). Among these genes, *VrOSCA1.4* showed the largest fold change in relative gene expression following treatment with the three types of osmotic stress than when under the normal growth conditions (Additional file 6). These results indicate that mung bean *OSCA* genes respond to osmotic stress caused by ABA, PEG and NaCl treatment.

### Cis-acting element analysis in the promoters of *VrOSCA* genes

**Fig. 6:**
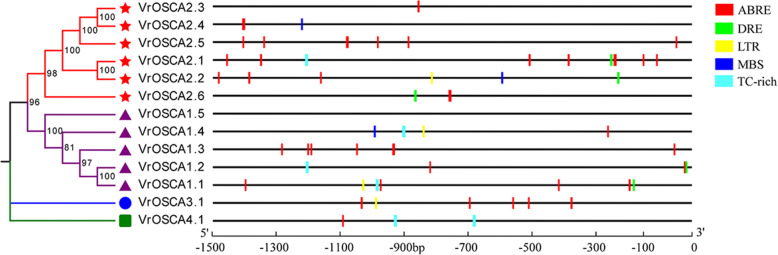
Distribution of the stress responsive cis-regulatory elements in the promoter regions of *VrOSCA* genes in mung bean. The ABRE, DRE, LTR, MBS and TC-rich sequences are represented by rectangles of different colors. ABRE: abscisic acid responsive element; DRE: drought, salt and cold responsive element; LTR: low temperature responsive element; MBS: drought responsive element; TC-rich: defense and stress responsive element

The cis-acting elements in promoter regions of genes participate in various pathways, for example, the ABA and abiotic stress response signal transduction pathways [31]. Therefore, we analyzed the cis-acting elements involved in ABA and abiotic stress responses in the 1.5 kb promoter region of *VrOSCA* genes, including ABRE, DRE, MBS, TC-rich and LTR elements. We found that all the *VrOSCA* genes, except *VrOSCA1.5*, contained at least one of these cis-acting elements (Fig. 6, Additional file 7). Moreover, the cis-acting elements of the *VrOSCA*s among clades were different. For example, clade 1 and clade 2 contained DRE and MBS elements associated with drought stress, but genes in clade 3 and clade 4 did not. Clade 1, clade 2 and clade 3 contained LTR elements associated with low temperature stress, while the gene in clade 4 did not (Fig. 6, Additional file 7). These results indicate that *VrOSCA* genes in different clades might respond to stress collectively. In clade 2, only *VrOSCA2.2* and *VrOSCA2.4* contained MBS element, only *VrOSCA2.1* contained TC-rich element, and only *VrOSCA2.2* contained LTR element (Fig. 6, Additional file 7). This phenomenon was also observed in clade 1. These results suggest that *VrOSCAs* in the same clade may have different functions.

## Discussion

Because mung bean is a broadly adapted and highly stress-tolerant crop, whole genome sequencing of mung bean is conducive to the identification of resistance genes and genetic improvement of crops. In the present study, we performed a genome-wide analysis of *OSCA* genes in mung bean and identified a total of 13 *VrOSCA* genes. The VrOSCA proteins varied substantially in sequence and physicochemical properties (Table 1), which were comparable with *OSCA* genes from other plant species [20, 24, 26, 32]. Phylogenetic tree (Fig. 1) analysis showed that OSCAs can be divided into four clades, which was consistent with evolutionary analysis of *Arabidopsis*, soybean and rice [20, 24, 26]. Each clade included OSCA members from mung bean, *Arabidopsis*, soybean and rice, indicating that the OSCA family originated and diversified prior to divergence of mung bean, *Arabidopsis*, soybean and rice. The clade 3 and clade 4 contained fewer members but were conserved across species, indicating that OSCA members in clade 3 and clade 4 may play an indispensable role in biological processes. The different numbers of OSCAs within the mung bean, *Arabidopsis*, soybean and rice genomes indicate that the majority of OSCAs in the mung bean, *Arabidopsis*, soybean and rice genomes undergo greater genetic variation after their divergence.

Based on OSCA family member phylogenetic relationships (Fig. 1), we systematically analyzed the synteny relationship of OSCAs in mung bean, *Arabidopsis*, soybean and rice (Fig. 2, Additional file 2). Large-scale expansion of *OSCAs* probably occurred after monocot and dicot division. Although *VrOSCA2.2* and *GmOSCA2.1* were clustered together (Fig. 1), *VrOSCA2.2* was absent from the synteny analysis. We did not find synteny blocks related to *VrOSCA1.2* and *VrOSCA2.2*. To elucidate the expanded mechanism of the *OSCA* gene family in mung bean, gene duplication events were investigated (Fig. 3, Table 2). We identified a total of 3 duplicated *VrOSCA* gene pairs, including *VrOSCA2.1/VrOSCA2.2*, *VrOSCA2.3/VrOSCA2.4* and *VrOSCA2.4/VrOSCA2.5*. The collinearity relationship between mung bean and soybean showed that *VrOSCA2.3, VrOSCA2.4* and *VrOSCA2.5* had collinearity relationships with *GmOSCAs*, but *VrOSCA2.2* did not (Additional file 2). Therefore, the duplication events of *VrOSCA2.1/VrOSCA2.2* might have occurred after the divergence of mung bean and soybean, while *VrOSCA2.3/VrOSCA2.4* and *VrOSCA2.4/VrOSCA2.5* duplicated prior to the divergence of mung bean and soybean. Ka/Ks ratios for the duplicated *VrOSCA* gene pairs were less than 1, suggesting that the duplicated *VrOSCAs* might have experienced purifying selective pressure (Table 2). As purifying selection restricts gene divergence, the duplicated *VrOSCA* genes might have retained some similar functions [33]. Our results also showed that the expression patterns of *VrOSCA2.3*, *− 2.4* and − 2.5 genes were similar under ABA-, PEG- and NaCl-induced osmotic stress.

Previous studies have shown that each AtOSCA protein contains 11 TMs [23, 34, 35]. In contrast, VrOSCAs contained 8–10 TMs, which indicates that VrOSCAs experienced genetic variation during evolution. To investigate the structural features of VrOSCAs, conserved domains were analyzed. The results showed that the structural domain was highly conserved (Fig. 4a) and the distribution of the pfam13967, pfam14703 and pfam02714 protein domains in VrOSCAs was consistent with that of the OSCA proteins in maize [32]. Meanwhile, all the TMs were in the pfam02714 and pfam13967 protein domains (Fig. 4a). In this study, 20 distinct conserved motifs were identified. The motifs of VrOSCAs in clade 1, clade 2 and clade 3 were highly conserved and the composition patterns of the conserved motifs in these three clades were similar. However, VrOSCA4.1 in clade 4 contained fewer conserved motifs than the other VrOSCAs (Fig. 4b). Moreover, the expression of *VrOSCA4.1* gene increased by a factor of less than 10-fold following treatment with ABA-, PEG- and NaCl-induced osmotic stress than when under the normal growth conditions (Additional file 6), suggesting that *VrOSCA4.1* may have an indirect function in the osmotic stress response [25].

In this study, the dynamic osmotic stress-responsive expression patterns of *VrOSCAs* were analyzed. Expression profile analysis of *VrOSCAs* can help us to understand their possible functions in osmotic stress and offer crucial clues for functional assessment. As members of the OSCA hyperosmotic calcium channel protein family, the *VrOSCA* genes responded to ABA-, PEG- and NaCl-induced osmotic stress, which is consistent with the response of *OSCA* genes in *Arabidopsis* and rice [24, 36]. However, *VrOSCAs* exhibited differential expression under osmotic stress, not only among clades but also among members within the same clade, suggesting that different *VrOSCAs* might have diverse functions. The present results showed that *VrOSCA2.1* was significantly downregulated by ABA, PEG and NaCl treatment, whereas the other 12 *VrOSCA* genes were significantly upregulated by these three types of osmotic stress (Fig. 5, Additional file 6), suggesting that the 12 *VrOSCAs* might be crucial mediators of the osmotic stress response and contribute to the establishment of complex signaling networks in mung bean. Upregulation of the 8 *VrOSCA* genes (except *VrOSCA1.1*, *− 1.2*, *− 1.3* and *− 4.1*) ranged from 10- to 70-fold (Additional file 6), which indicated that these genes responded positively to osmotic stress. *VrOSCA2.2* and *− 2.4* responded strongly to ABA, PEG and NaCl stress and showed increased expression of more than 20-fold when compared to the expression levels under control conditions (0 h) (Additional file 6). Thus, *VrOSCA2.2* and *− 2.4* may simultaneously respond to ABA, PEG and NaCl stress-response pathways, and there may be interactions in the pathways that are responsive to the three types of osmotic stress. Regardless, these genes played an important role in drought and high-salinity tolerance. Moreover, the expression of duplicated genes showed that two pairs of duplicated genes shared similar expression patterns, suggesting that the genes might have retained some essential functions during subsequent evolution. However, the duplicated genes *VrOSCA2.1*/*VrOSCA2.2* showed divergent expression, which might have experienced functionalization after the duplication events [37]. Our work has identified genes for further characterization of their functional involvement in osmotic stress.

Analysis of promoter components of the 13 *VrOSCA* genes revealed variable types of core components associated with ABA responsiveness (ABRE) and stress responsiveness (DRE, MBS, LTR and TC-rich). For example, all the genes contained ABRE core components that play a crucial role in ABA-dependent gene expression, except *VrOSCA1.5*. Only five *VrOSCA* genes, *VrOSCA1.1*, *− 1.2*, *− 2.1*, *− 1.2* and*-2.6*, contained DRE element (Fig. 6, Additional file 7). Furthermore, the promoters of *VrOSCA* genes classified in the same clade also contained different types and numbers of response elements. Therefore, different genes classified in the same clade may show functional diversity and may also have different mechanisms of action [38]. In clade 1, only *VrOSCA1.4* contained MBS element associated with the drought response (Fig. 6, Additional file 7). The relative expression value of *VrOSCA1.4* was significantly higher than other *VrOSCAs* in clade 1 under PEG treatment (Additional file 6), suggesting that *VrOSCA1.4* may play a more important role in the response to drought stress. In addition, the promoters of *VrOSCA2.2* and *− 2.4* also contained MBS elements (Fig. 6). The relative expression level of *VrOSCA2.2* and *− 2.4* increased more than 20-fold compared with the control (0 h) under PEG treatment (Additional file 6). This result indicated that genes in different clades may exhibit synergies [39]. The promoters of *VrOSCA2.2* and *− 2.6* contained ABRE (abscisic acid responsive element) and DRE (drought, salt and cold responsive element) elements, and the relative expression levels of *VrOSCA2.2* and *− 2.6* were more than 10-fold that of the control (0 h) under ABA, PEG and NaCl stress (Additional file 6). Therefore, the stress-inducible cis-acting regulatory elements present in the promoter play an important role in modulating the expression of genes in response to abiotic stress.

## Conclusions

In conclusion, a total of 13 *OSCA* genes were identified in mung bean. The comprehensive analysis of the VrOSCA gene family provided important information such as phylogenetic relationships, duplication events and expansion profiles. These findings provide an important foundation for understanding the molecular evolution of the OSCA family in mung bean and provide candidate genes for further study of abiotic stress tolerance in mung bean.

## Methods

### Identification of OSCA gene family members in the mung bean genome

The *V. radiata* genome database (genome assembly: Vradiata_ver6) was downloaded from EnsemblPlants (http://plants.ensembl.org/index.html). The conserved OSCA DUF221 protein domain (pfam accession number: 02714) from the Pfam database [40] was used to build the HMM profiles (http://hmmer.janelia.org/) and query the *V. radiata* whole-genome protein database. Each non-redundant sequence was confirmed using the Simple Modular Architecture Research Tool (SMART) web server (http://smart.embl.de/) [41], the Conserved Domain Database (CDD) in the National Center for Biotechnology Information (NCBI) (https://www.ncbi.nlm.nih.gov/Structure/bwrpsb/bwrpsb.cgi) [42] and the Pfam website (http://pfam.xfam.org/). The MW and pI of OSCA proteins were predicted with ProtParam (http://web.expasy.org/protparam/).

### Conserved motifs, TMs and phylogenetic analysis

The conserved motifs and TMs of mung bean OSCA proteins were identified using the MEME program (http://meme-suite.org/meme/) and the TMHMM Server V.2.0 (www.cbs.dtu.dk/services/TMHMM/), respectively. Multiple sequence alignment was analyzed with the ClustalW program [43], and the phylogenetic tree was constructed using MEGA 7 (Molecular Evolutionary Genetics Analysis) software with the neighbor-joining (NJ) method and 1000 replicate iterations [44].

### Interspecies synteny analysis and gene duplication

To analyze the relationships of orthologous *OSCA* genes in different species, multiple sequence alignment was used to detect the sequences of mung bean and other species with a similarity of more than 70%. Then, the Multiple Collinearity Scan toolkit (MCScanX) was adopted to analyze the collinear block with the default parameters. Finally, the linear analysis map was illustrated using Dual Synteny Plotter software (https://github.com/CJ-Chen/TBtools). Duplicated gene pairs were analyzed using the MCScanX program with the default parameters and plotted with Circos software [45]. The Ka (nonsynonymous substitution rate) and Ks (synonymous substitution rate) were investigated by DnaSP v5.0 software [46], and the selection pressure was calculated by the Ka/Ks ratio.

### Plant materials and stress treatments

In this study, the mung bean cultivar VC1973A was used to analyze gene expression profiles following treatment with drought, salt and ABA. The seeds of cultivar VC1973A were obtained from the Chinese Academy of Agricultural Sciences. VC1973A was grown in a growth chamber at 24 °C with a photoperiod of 16 h. When the first trifoliolate leaf appeared, seedlings were treated with 20% PEG-6000, NaCl (100 mM) and ABA (100 μM) solution as described previously [47]. The leaves were collected at 0 h, 4 h, 12 h and 24 h and stored at − 80 °C.

### Expression profile analysis of *VrOSCA* genes under stress treatments

Total RNA from the leaves was isolated using an RNAprep Pure Plant Kit (Tiangen, Beijing, China), and first-strand cDNA was synthesized using a SuperScript™ III Reverse Transcriptase kit (Invitrogen, USA). Quantitative real-time PCR (qRT-PCR) was performed in an ABIViiA 7 Real-Time PCR system (Applied Biosystems, USA) with SYBR Green PCR mix (QIAGEN). PCR was performed with the following conditions: 95 °C for 2 min followed by 40 cycles of 94 °C for 10 s and 59 °C for 10 s. The relative expression level of *VrOSCA* genes was calculated by the 2^-∆∆CT^ method [48]. Gene-specific primers were designed using Primer Express Software v2.0 (Additional file 8) and synthesized commercially (HUADA Gene, Beijing, China). The *V. radiata* actin gene (GenBank: AF143208.1) was used as an endogenous control for qRT-PCR. Each experiment was repeated using different cDNAs from three biological replicates. The heatmap of *VrOSCA* gene expression was generated using TBtools (v0.6652) and was clustered hierarchically based on the expression patterns. For statistical convenience, log2 expression values were used for the expression of *VrOSCAs* in the heatmap.

### Abiotic stress-responsive cis-regulatory element analysis in the promoter regions of *VrOSCA* genes

The sequences of 1.5 kb promoter regions upstream of *VrOSCA* genes were downloaded from EnsemblPlants (http://plants.ensembl.org/index.html) (Additional file 9). The PLACE website (http://www.dna.affrc.go.jp/PLACE/?action=newplace) [49] was used to identify the putative cis-regulatory elements involved in ABA and abiotic stress responses in the promoter region.

## Supplementary Information

**Additional file 1.**

**Additional file 2.**

**Additional file 3.**

**Additional file 4.**

**Additional file 5.**

**Additional file 6.**

**Additional file 7.**

**Additional file 8.**

**Additional file 9.**

## Data Availability

The *Arabidopsis* OSCA protein sequences were collected from the *Arabidopsis* information source (TAIR) database (http://www.arabidopsis.org). The rice and soybean OSCA protein sequences were obtained from the EnsemblPlants (http://plants.ensembl.org/index.html). All the accession numbers of OSCAs were contained in the additional file 1. The genome sequences of mung bean (Vradiata_ver6), *soybean (Glycine_max_v2.1)* and rice (IRGSP-1.0) were downloaded from EnsemblPlants (http://plants.ensembl.org/index.html). All the datasets used and analyzed during the current study are included in the published article and its additional files.

## Abbreviations

OSCA: Hyperosmolality-gated calcium-permeable channels
TMs: Transmembrane regions
SMART: Simple modular architecture research tool
NCBI: National center for biotechnology information
TAIR: The arabidopsis information resource
CDD: Conserved domain database
MW: Molecular weight
pI: Isoelectric point
MEGA: Molecular evolutionary genetics analysis
MEME: Multiple EM for motif elicitation
Ka/Ks: Non-synonymous substitution rate/synonymous substitution rate
qRT-PCR: Quantitative real-time PCR

## References

[1] Ahanger MA, Alyemeni MN, Wijaya L, Alamri SA, Alam P, Ashraf M, Ahmad P (2018). Potential of exogenously sourced kinetin in protecting Solanum lycopersicum from NaCl-induced oxidative stress through up-regulation of the antioxidant system, ascorbate-glutathione cycle and glyoxalase system. PLoS One.

[2] Huang Z, Tang J, Duan W, Wang Z, Song X, Hou X (2015). Molecular evolution, characterization, and expression analysis of SnRK2 gene family in Pak-choi (Brassica rapa ssp. chinensis). Front. Plant Sci.

[3] Kaya C, Senbayram M, Akram NA, Ashraf M, Alyemeni MN, Ahmad P (2020). Sulfur-enriched leonardite and humic acid soil amendments enhance tolerance to drought and phosphorus deficiency stress in maize (Zea mays L.). Sci Rep.

[4] Ahanger MA, Aziz U, Alsahli AA, Alyemeni MN, Ahmad P (2019). Influence of exogenous salicylic acid and nitric oxide on growth, photosynthesis, and ascorbate-glutathione cycle in salt stressed Vigna angularis. Biomolecules..

[5] Alam P, Albalawi TH, Altalayan FH, Bakht MA, Ahanger MA, Raja V, Ashraf M, Ahmad P (2019). 24-Epibrassinolide (EBR) confers tolerance against NaCl stress in soybean plants by up-regulating antioxidant system, ascorbate-glutathione cycle, and glyoxalase system. Biomolecules..

[6] Bartels D, Sunkar R (2005). Drought and salt tolerance in plants. Crit Rev Plant Sci.

[7] McAinsh MR, Pittman JK (2009). Shaping the calcium signature. New Phytol.

[8] Kaur H, Sirhindi G, Bhardwaj R, Alyemeni MN, Siddique KHM, Ahmad P (2018). 28-homobrassinolide regulates antioxidant enzyme activities and gene expression in response to salt- and temperature-induced oxidative stress in Brassica juncea. Sci Rep.

[9] Raja V, Qadir SU, Alyemeni MN, Ahmad P (2020). Impact of drought and heat stress individually and in combination on physio-biochemical parameters, antioxidant responses, and gene expression in Solanum lycopersicum. 3 Biotech.

[10] Farooq A, Bukhari SA, Akram NA, Ashraf M, Wijaya L, Alyemeni MN, Ahmad P (2020). Exogenously applied ascorbic acid-mediated changes in osmoprotection and oxidative defense system enhanced water stress tolerance in different cultivars of safflower (Carthamus tinctorious L.). Plants.

[11] Begum N, Ahanger MA, Su Y, Lei Y, Mustafa NSA, Ahmad P, Zhang L (2019). Improved drought tolerance by AMF inoculation in maize (Zea mays) involves physiological and biochemical implications. Plants..

[12] Hepler PK (2005). Calcium: a central regulator of plant growth and development. Plant Cell.

[13] Reddy AS (2001). Calcium: silver bullet in signaling. Plant Sci.

[14] Hubbard KE, Siegel RS, Valerio G, Brandt B, Schroeder JI (2012). Abscisic acid and CO_2_ signalling via calcium sensitivity priming in guard cells, new CDPK mutant phenotypes and a method for improved resolution of stomatal stimulus-response analyses. Ann Bot.

[15] Knight H, Trewavas AJ, Knight MR (1997). Calcium signalling in Arabidopsis thaliana responding to drought and salinity. Plant J.

[16] Steinhorst L, Kudla J (2013). Calcium - a central regulator of pollen germination and tube growth. Biochim Biophys Acta.

[17] Booth IR, Edwards MD, Black S, Schumann U, Miller S (2007). Mechanosensitive channels in bacteria: signs of closure?. Nat Rev Microbiol.

[18] Arnadóttir J, Chalfie M (2010). Eukaryotic mechanosensitive channels. Annu Rev Biophys.

[19] Batistič O, Kudla J (2012). Analysis of calcium signaling pathways in plants. Biochim Biophys Acta.

[20] Yuan F, Yang H, Xue Y, Kong D, Ye R, Li C, Zhang J, Theprungsirikul L, Shrift T, Krichilsky B, Johnson DM, Swift GB, He Y, Siedow JN, Pei ZM (2014). OSCA1 mediates osmotic-stress-evoked Ca^2+^ increases vital for osmosensing in Arabidopsis. Nature..

[21] Moeder W, Phan V, Yoshioka K (2019). Ca^2+^ to the rescue - Ca^2+^ channels and signaling in plant immunity. Plant Sci.

[22] Hou C, Tian W, Kleist T, He K, Garcia V, Bai F, Hao Y, Luan S, Li L (2014). DUF221 proteins are a family of osmosensitive calcium-permeable cation channels conserved across eukaryotes. Cell Res.

[23] Liu X, Wang J, Sun L (2018). Structure of the hyperosmolality-gated calcium-permeable channel OSCA1.2. Nat Commun.

[24] Li Y, Yuan F, Wen Z, Li Y, Wang F, Zhu T, Zhuo W, Jin X, Wang Y, Zhao H, Pei ZM, Han S (2015). Genome-wide survey and expression analysis of the OSCA gene family in rice. BMC Plant Biol.

[25] Cao L, Zhang P, Lu X, Wang G, Wang Z, Zhang Q, et al. Systematic analysis of the maize OSCA genes revealing ZmOSCAfamily members involved in osmotic stress and ZmOSCA2.4 confers enhanced drought tolerance in transgenic Arabidopsis. Int J Mol Sci. 2020;21(1):351. 10.3390/ijms21010351 PMC6982122 31948071

[26] Li JW, Yang JK, Jia BW (2017). Evolution and expression analysis of OSCA gene family in soybean. Chin J Oil Crop Sci.

[27] Kim SK, Nair RM, Lee J, Lee SH (2015). Genomic resources in mungbean for future breeding programs. Front Plant Sci.

[28] Kang YJ, Kim SK, Kim MY, Lestari P, Kim KH, Ha BK, Jun TH, Hwang WJ, Lee T, Lee J, Shim S, Yoon MY, Jang YE, Han KS, Taeprayoon P, Yoon N, Somta P, Tanya P, Kim KS, Gwag JG, Moon JK, Lee YH, Park BS, Bombarely A, Doyle JJ, Jackson SA, Schafleitner R, Srinives P, Varshney RK, Lee SH (2014). Genome sequence of mungbean and insights into evolution within Vigna species. Nat Commun.

[29] Wang W, Vinocur B, Altman A (2003). Plant responses to drought, salinity and extreme temperatures: towards genetic engineering for stress tolerance. Planta..

[30] Finkelstein R (2013). Abscisic acid synthesis and response. Arabidopsis Book.

[31] Ji L, Wang J, Ye M, Li Y, Guo B, Chen Z, Li H, An X (2013). Identification and characterization of the Populus AREB/ABF subfamily. J Integr Plant Biol.

[32] Ding S, Feng X, Du H, Wang H. Genome-wide analysis of maize OSCA family members and their involvement in drought stress. Peer J. 2019;7:e6765. 10.7717/peerj.6765 PMC6462396 30997296

[33] Liu W, Li W, He Q, Daud MK, Chen J, Zhu S (2014). Genome-wide survey and expression analysis of calcium-dependent protein kinase in Gossypium raimondii. PLoS One.

[34] Jojoa-Cruz S, Saotome K, Murthy SE, Tsui CCA, Sansom MS, Patapoutian A, Ward AB (2018). Cryo-EM structure of the mechanically activated ion channel OSCA1.2. Elife.

[35] Zhang M, Wang D, Kang Y, Wu JX, Yao F, Pan C, Yan Z, Song C, Chen L (2018). Structure of the mechanosensitive OSCA channels. Nat Struct Mol Biol.

[36] Kiyosue T, Yamaguchi-Shinozaki K, Shinozaki K (1994). Cloning of cDNAs for genes that are early-responsive to dehydration stress (ERDs) in Arabidopsis thaliana L.: identification of three ERDs as HSP cognate genes. Plant Mol Biol.

[37] Adams KL (2007). Evolution of duplicate gene expression in polyploid and hybrid plants. J Hered.

[38] Yoshida T, Mogami J, Yamaguchi-Shinozaki K (2014). ABA-dependent and ABA-independent signaling in response to osmotic stress in plants. Curr Opin Plant Biol.

[39] Fujita Y, Fujita M, Shinozaki K, Yamaguchi-Shinozaki K (2011). ABA-mediated transcriptional regulation in response to osmotic stress in plants. J Plant Res.

[40] Finn RD, Bateman A, Clements J, Coggill P, Eberhardt RY, Eddy SR, Heger A, Hetherington K, Holm L, Mistry J, Sonnhammer ELL, Tate J, Punta M (2014). Pfam: the protein families database. Nucleic Acids Res.

[41] Letunic I, Doerks T, Bork P (2015). SMART: recent updates, new developments and status in 2015. Nucleic Acids Res.

[42] Marchler-Bauer A, Lu S, Anderson JB, Chitsaz F, Derbyshire MK, DeWeese-Scott C, Fong JH, Geer LY, Geer RC, Gonzales NR, Gwadz M, Hurwitz DI, Jackson JD, Ke Z, Lanczycki CJ, Lu F, Marchler GH, Mullokandov M, Omelchenko MV, Robertson CL, Song JS, Thanki N, Yamashita RA, Zhang D, Zhang N, Zheng C, Bryant SH (2011). CDD: a conserved domain database for the functional annotation of proteins. Nucleic Acids Res.

[43] Thompson JD, Higgins DG, Gibson TJ (1994). CLUSTAL W: improving the sensitivity of progressive multiple sequence alignment through sequence weighting, position-specific gap penalties and weight matrix choice. Nucleic Acids Res.

[44] Kumar S, Stecher G, Tamura K (2016). MEGA7: molecular evolutionary genetics analysis version 7.0 for bigger datasets. Mol Biol Evol.

[45] Krzywinski M, Schein J, Birol I, Connors J, Gascoyne R, Horsman D, Jones SJ, Marra MA (2009). Circos: an information aesthetic for comparative genomics. Genome Res.

[46] Librado P, Rozas J (2009). DnaSP v5: a software for comprehensive analysis of DNA polymorphism data. Bioinformatics..

[47] Chung E, Cho CW, So HA, Kang JS, Chung YS, Lee JH (2013). Overexpression of VrUBC1, a mung bean E2 ubiquitin-conjugating enzyme, enhances osmotic stress tolerance in Arabidopsis. PLoS One.

[48] Livak KJ, Schmittgen TD (2001). Analysis of relative gene expression data using real-time quantitative PCR and the 2(−Delta Delta C(T)) method. Methods..

[49] Lescot M, Déhais P, Thijs G, Marchal K, Moreau Y, Van de Peer Y, Rouzé P, Rombauts S (2002). PlantCARE, a database of plant cis-acting regulatory elements and a portal to tools for in silico analysis of promoter sequences. Nucleic Acids Res.

